# Hemoglobin Precipitation: An Index of *In Vitro* Vasoconstrictive Activities of Methanol Leaf Extracts of C*roton megalocarpus* Hutch and *Lantana camara* Linn

**DOI:** 10.1155/2021/3817106

**Published:** 2021-11-28

**Authors:** Hezron Mutisya Muindi, Cromwell Mwiti Kibiti, Mathew Piero Ngugi

**Affiliations:** ^1^Department of Biochemistry, Microbiology & Biotechnology, School of Pure and Applied Sciences, Kenyatta University, P.O. Box 43844-00100, Nairobi, Kenya; ^2^Department of Pure and Applied Sciences, Technical University of Mombasa, P.O. Box 90420-80100, Mombasa, Kenya

## Abstract

The function of innate hemostasis aids the body in bleeding control, preventing the loss of excessive amounts of blood following low-degree injuries. However, injuries of a higher degree may require extrinsic intervention to stop life-threatening blood loss. Astringent agents' actions result in mechanical constriction of small blood vessels and shrinkage of body tissues, thereby stopping blood loss. This enhances the primary phase of hemostasis, where vasoconstriction is the main mechanism at play during the initial response to injury. The effects of plant extracts on protein precipitation have been linked to blood vessel vasoconstriction. Traditionally, the leaves of *Croton megalocarpus* Hutch and *Lantana camara* Linn plants are used by communities living in Makueni County, Kenya, for peripheral bleeding control. However, the effects of extracts of both plants on hemoglobin precipitation have not been evaluated scientifically. In the current study, the activities of methanol extracts of *C. megalocarpus* (H.) and *L. camara* (L.) on blood protein precipitation were investigated. The leaves were harvested, cleaned, air-dried, milled, and extracted in absolute methanol before being concentrated into dry powders. A qualitative phytochemical screen revealed the presence of terpenoids, steroids, tannins, phenols, flavonoids, reducing sugars, cardiac glycosides, and carbohydrates in the methanol extract of *C. megalocarpus* (H.). The methanol extracts of *L. camara* (L.) contained cardiac glycosides, saponins, tannins, phenols, terpenoids, reducing sugars, and carbohydrates. The hemoglobin precipitation ability of various concentrations of extracts using mice samples was presented as relative astringency following the tannic acid external standard method. Methanol extracts *C. megalocarpus* (H.) and *L. camara* (L.) had significantly higher relative astringency compared with the normal control, indicating a protein precipitating activity. The relative astringency observed in both plant extracts is linked to the activity of tannins, phenols, flavonoids, and saponins detected during preliminary phytochemical screening.

## 1. Introduction

Primary and secondary phases of hemostasis are initiated simultaneously following the breach of blood vessel integrity [1, 2]. Primary hemostasis results in vascular constriction at the site of injury, reducing blood loss mechanically [1]. Platelets are then activated and attracted to the site due to the effect of Von Willebrand factor, which mediates the cells' binding to vessel collagen, and to each other to form a temporary plug [1, 3]. The function of secondary hemostasis is to reinforce the temporary platelet plug formed at the end of primary hemostasis, by forming a fibrin meshwork over the site [2]. Secondary hemostasis involves intrinsic, extrinsic, and common pathways consisting of a series of enzymes, minerals, and associated proteins [3, 4]. Major injuries may overwhelm the coagulation system, leading to a profound loss of blood, which is associated with life-threatening conditions such as anemia, shock, and ischemia [3].

The control of bleeding resulting from vascular injury is important for survival and good health [2]. Astringent agents are a group of substances whose effects on an injury site result in the constriction of small blood vessels and shrinkage of tissues [5, 6]. Conventional astringents which are commonly used include epinephrine, aluminum sulfate, aluminum chloride, ferric sulfate, zinc sulfate, and zinc chloride [5–7]. Current astringents used are associated with a number of unwanted effects. Aluminum sulfate affects eugenol cement setting reactions when the astringent is used in dentistry [5, 7]. Aluminum chloride, calcium sulfate, and zinc chloride cause pain at the site of application [5]. Aluminium chloride and ferric chloride cause local tissue destruction [7].

The world has witnessed a surge in interest in plant-based therapy due to perceived safety from side effects and the assumption that natural products are harmless [8]. However, the safety of herbal remedies needs assessment since the reliance on traditional beliefs and assumptions in medicine is discouraged [9]. Traditionally, there are many plant materials that are used in the control of injury-related blood loss in humans. Sap from the fresh leaf petiole of *Croton megalocarpus* Hutch is used topically to arrest bleeding resulting from minor injuries and for the treatment of wounds on the skin [10]. Paste from crushed fresh leaves of *Lantana camara* Linn is widely used to control bleeding and as an antiseptic when it is applied over fresh cuts and wounds [11–13]. The same materials from both plants are applied topically to injured skin to stop bleeding by communities living in Makueni County in South Eastern Kenya. These traditional uses have not been confirmed and/or validated scientifically. The aim of this study was to evaluate the ability of methanolic leaf extracts of *C. megalocarpus* (H.) and *L. camara* (L.) to precipitate hemoglobin, a red blood cell protein.

## 2. Materials and Methods

### 2.1. Collection of Plant Materials

With the help from a local herbalist, fresh leaves of *C. megalocarpus* (H.) and *L. camara* (L.) were harvested in the wild from Mbooni subcounty in Makueni, Southeast Kenya. The GPS coordinates for locations where *C*. *megalocarpus* (H.) and *L. camara* (L.) leaves were harvested for this study are 1°35′28.7″S, 37°25′55.6″E and 1°35′27.1″S, 37°25′59.2″E, respectively. The leaves were transported in plastic bags to the Biochemistry Laboratory at the Kenya Methodist University School of Medicine and Health Sciences, Meru campus. The leaves were cleaned in water and air-dried under shade for 14 days.

Samples of the plant consisting of attached stems, leaves, and flowers were spread, covered with newspaper, and pressed using herbarium frames for 14 days. The samples were later mounted on stiff but flexible paper and later deposited for plant identification and voucher number acquisition. The samples were identified visually and labeled accordingly by a resident taxonomist, and voucher specimens were archived at the United States International University, Africa's School of Pharmacy and Health Sciences herbarium. Voucher numbers MUINDI.H/014 for *C. megalocarpus* (H.) and MUINDI.H/013 for *L. camara* (L.) are available for future reference and verification.

### 2.2. Extract Preparation

Dry leaves were ground into powder using an electric mill (Christy and Norris Model 8). The powders were weighed separately using an analytical balance (Citizen Scales, Edison, USA) and placed in clean beakers and then mixed with absolute methanol (ratio of 150 grams of leave powder to 1.5 liters of methanol). An extract of the blended leaves of both plants was made by mixing powders (75 g of *C. megalocarpus* (H.) and an equal weight of *L. camara* (L.)) in a beaker with 1.5 liters of absolute methanol. The beakers with the mixtures were agitated continuously on a rocker mixer for 48 hours, before they were decanted and filtered using a Buchner funnel apparatus with Whatman paper. The filtrates were concentrated into dry powders using a rotary evaporator (Stuart RE400, USA).

### 2.3. Extract Solutions and Control Preparation for Analysis

Methanol extracts of *C. megalocarpus* (H.), *L. camara* (L.), and *C. megalocarpus* (H.)-*L. camara* (L.) blend were weighed using an analytical balance (Citizen Scales, Edison, USA) and dissolved in distilled water to make final concentrations of 0.25, 0.5, and 1 mg/ml. To enhance solubility, a volume of 0.1 ml of dimethyl sulfoxide (DMSO) was added to each extract solution. Ivyzinc™, a commercial astringent (Ivee Aqua EPZ Limited, Kenya), a reference astringent containing zinc sulfate solution, was used at a neat concentration of 1 mg/ml.

### 2.4. Preparation of Tannic Acid External Standard Solution

Tannic acid standards were made by weighing powder using a Citizen CY-204 analytical balance and dissolving it in distilled water in volumetric flasks to make concentrations of 0.25, 0.50, 0.75, 1, 2, 4, and 8 mg/ml.

### 2.5. Blood Collection and Preparation

Authority to use laboratory animals was granted by the National Commission for Science, Technology, and Innovation (reference number NACOSTI/P/20/4729). Blood was collected from 55 laboratory bred adult mice (Swiss white albino) by cardiac puncture into plastic tubes with sodium citrate anticoagulant (Vacutainer, BD). The blood samples were centrifuged using an Eppendorf 5427R (Eppendorf, Germany) for 5 minutes at 7000 rpm. Packed blood was separated from plasma and stored under refrigeration awaiting evaluation.

### 2.6. Pooled Blood Hemolysate Preparation

A pooled packed blood cell aliquot for linearity tannic acid standard evaluation was prepared by mixing 100 µL of each of 10 randomly selected mouse blood samples using an Eppendorf micropipette (Eppendorf, Germany) into a sterile plastic container. Hemolysate of pooled blood was prepared by measuring 100 *µ*L of mixed samples using an Eppendorf micropipette into 5 ml of distilled water. The hemolysate was allowed to stand for 5 minutes before evaluation.

### 2.7. Sample Hemolysate Preparation

Hemolysates were prepared by measuring 100 *µ*L of the respective packed blood sample using an Eppendorf micropipette and then mixing it with 5 ml of distilled water. The preparations were allowed to stand for 5 minutes before evaluation to facilitate complete hemolysis.

### 2.8. Experimental Design

The protein precipitation study for astringency followed a controlled experimental design. Astringency was evaluated using the spectrophotometric hemoglobin precipitation-tannic acid external standard method by Ferreiraa et al., (2003) in which Bate-Smith (1973) was cited. Blood samples collected from 55 mice by cardiac puncture were randomly placed into 11 treatment groups. Blood samples were mixed with distilled water and allowed to stand for five minutes for complete hemolysis of red blood cells. The hemolysates were mixed with methanol leaf extract of *C. megalocarpus* (H.), *L. camara* (L.) and that of *C. megalocarpus* (H.)-*L. camara (*L.) blend at concentrations of 0.25, 0.5, and 1 mg/ml. A reference drug (Ivyzinc™) was evaluated at 1 mg/ml, while distilled water was used as the negative control.

The hemolysate-extract-control mixtures were allowed to stand for a few minutes before centrifugation to allow for possible precipitation. The optical density (OD) of the supernatant obtained upon centrifugation and separation was determined. The ODs of all samples and controls were extrapolated onto the tannic acid standard curve, and the corresponding tannic acid equivalent (TAE) was determined and recorded. The TAE was used to calculate the relative astringency by dividing the concentration of the extract/control by the corresponding TAE.

### 2.9. Tannic Acid Standard Evaluation

Using an Eppendorf pipette, 2.5 ml of standard tannic acid solutions at concentrations of 0.25, 0.50, 0.75, 1, 2, 4, and 8 mg/ml were measured and placed separately in labeled glass test tubes. In each of the test tubes, 2.5 ml of hemolysate from a pooled blood sample was added and mixed and then allowed to stand for 15 minutes. The preparation was centrifuged at 7000 rpm for 5 minutes on a Hettich machine (Andreas Hettich GmbH and Co. KG, Germany). The supernatant was transferred into clean, labeled test tubes using a Pasteur pipette, and the optical density (OD) was read at 578 nm using a Shimadzu UV-Vis spectrophotometer model UV-1280 (Shimadzu, Japan). The OD obtained at various concentrations of tannic acid was plotted on a standard curve, and then a line of best fit was determined.

### 2.10. Evaluation of Protein Precipitation

A volume of 2.5 ml of blood hemolysate was placed into a clean, labeled glass test tube. An equal volume of the respective extract (or control) solution is added into the test tube containing the blood hemolysate. The hemolysate extract (or control) mixtures were allowed to stand for 15 minutes at room temperature before centrifugation using a Hettich machine (Andreas Hettich GmbH and Co. KG, Germany) at 7000 rpm for 5 minutes. The supernatant from each preparation was separated into clean, labeled test tubes, and their absorbance was measured at 578 nm using a Shimadzu UV-VIS spectrophotometer model UV-1280. The test was done thrice for each blood sample. The average absorbance was calculated, and the results were recorded.

### 2.11. Relative Astringency Calculation

The optical density (OD) results obtained for each treatment and sample were compared to the matching OD of tannic acid on the standard curve to determine the tannic acid concentration equivalent. The relative astringency was calculated using (1) described by [15] as follows:

(1) $$\begin{array}{c} \text{relative Astringency}\left(\text{RA}\right)=\frac{\text{concentration of extract or control}}{\text{tannic acid equivalent}}. \end{array}$$

### 2.12. Qualitative Phytochemical Analysis

The qualitative phytochemical analysis of methanol extract of *Croton megalocarpus* and *L. camara* was done following the standard methods as described by Ajuru et al. (2017).

The ethanol-ferric chloride test for phenol was done by mixing 25 g of the extracts (dissolved separately in 2 ml of water) with 2 ml of a combination of equal volumes of ethanol and distilled water. A drop of dilute ferric chloride solution was then added to the mixture. A blue color development indicated the presence of phenols.

The Salkowski test for steroids involved dissolving the extracts in a volume of 0.5 ml of distilled water. A combination of chloroform (1 ml) and a few drops of sulphuric acid was then added. The mixture was shaken and allowed to stand for about 3 minutes. A red-brown color at the interface of the components indicated the presence of steroids.

The Salkowski test for terpenoids involved mixing 1 ml of chloroform containing a few drops of sulphuric acid with solutions of extracts (0.25 grams of the extracts in 1 ml of distilled water). The mixture was shaken and allowed to stand for about three minutes. The reddish-brown coloration at the interface confirmed the presence of terpenoids.

The biuret method test for proteins was done by separating the extract solution (0.5 grams in 1 ml of distilled water) into two test tubes with 0.5 ml each. Sodium hydroxide solution (0.5 ml) was added and mixed. Copper sulfate solution (10 drops) was then added to the mixture and shaken. The absence of purple color formation confirmed the absence of proteins.

The ferric chloride test for tannins involved stirring 0.5 g of the extracts separately in beakers with 1 ml of distilled water and then filtering. Aqueous ferric chloride (1 ml of 15% w/v) was added to the filtrate. A green precipitate indicated a positive test.

Meyer's test for alkaloids involved heating a mixture of 0.25 g of the extract and 2.5 ml of 10% sulphuric acid in a boiling water bath for 5 minutes. Two drops of Meyer's reagent were added into the hot solution. A white precipitate confirmed the presence of alkaloids.

Molisch's test for carbohydrates was done by adding 5 ml of Molisch's reagent in plant extract solutions (0.25 grams of the extract and 1 ml of distilled water) in a test tube. Concentrated sulphuric acid (0.5 ml) was layered. The presence of a purple, red, and violet color at the liquid interface after 3 minutes confirmed the presence of carbohydrates.

The froth method was performed to test for saponins in the plant extracts. In test tubes containing 2.5 ml of distilled water, 0.25 grams of the extract were added and shaken vigorously before the test tubes were allowed to stand for 5 minutes. The presence of a persistent froth was suggestive of saponins. The formation of a soluble emulsion upon the addition of a few drops of olive oil confirmed the presence of saponins.

The Keller Killiani test for cardiac glycosides was done by dissolving 0.25 grams of the extracts in 1 ml of distilled water in test tubes before the addition of glacial acetic acid (0.5 ml) containing a few drops of ferric chloride. Sulphuric acid (concentrated, 0.5 ml) was layered carefully on the mixture. A brown ring at the interface confirmed the presence of cardiac glycosides.

The ammonium test for anthraquinones involved mixing 0.25 g of the extracts with 2.5 ml of chloroform in beakers. The mixture was filtered into clean test tubes. Ammonium solution (10% concentration, 2.5 ml) was added to the mixture and shaken. A pink-red brown color in the layer of the interface confirmed a negative test for anthraquinones.

Fehling's test for reducing sugar involved mixing 1 ml of Fehling's ‘A' with 1 ml of Fehling's ‘B' solution in a separate beaker. Fehling's solution mixture (1 ml) was combined with extract solution (0.25 grams in 1 ml of distilled water) and was dissolved separately in test tubes. An orange-red precipitate confirmed the presence of reducing sugar.

A lead acetate test for flavonoids was carried out by dissolving 0.25 grams of the extracts in 1 ml of distilled water in clean test tubes. A solution of lead acetate (10%) was added to the extract solution. A white precipitate indicated the presence of flavonoids.

### 2.13. Data Analysis

Quantitative data from the relative astringency determination was entered into a spreadsheet in MS Excel and cleaned. The data were exported to Minitab version 19.0 (State College, Pennsylvania) and subjected to descriptive statistics and expressed in mean ± SEM. Analysis was done using one-way ANOVA followed by Tukey's post hoc test for pairwise comparison and separation means. The values of *p* ≤ 0.05 were considered significant.

## 3. Results

### 3.1. Tannic Acid Standard Curve

**Figure 1:**
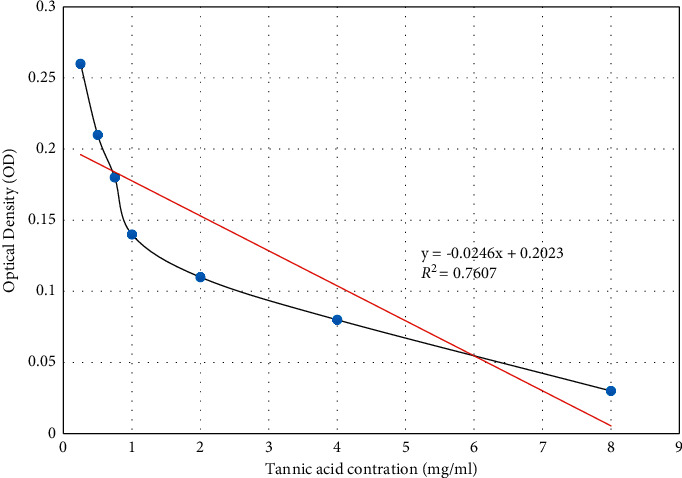
Tannic acid astringency standard curve. *In vitro* effects of methanol extracts of *C. megalocarpus* (H.) and *L. camara* (L.) on hemoglobin precipitation.

The graph reveals an inverse, dose-dependent relationship where a higher concentration of the standard corresponded to a lower optical density (OD), which in turn represented a higher hemoglobin precipitation activity, as shown in Figure 1. The data were used to derive a line of best fit and a linear equation for the graph.

**Figure 2:**
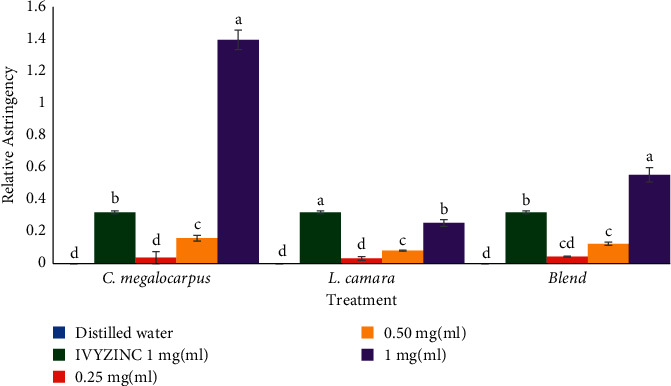
Relative astringency of MeCM, MeLC, and MeCM-MeLC extracts. MeCM (methanol leaf extract of *Croton megalocarpus*), MeLC (methanol leaf extract of *Lantana camara*), and MeCM-MeLC (methanol *Croton megalocarpus* and *Lantana camara* blended leaf extract). Each bar graph in the respective group represents 5 separate sample runs (*n* = 55) and is expressed as the mean ± SEM of the dataset. Error bars represent the standard error of mean measurement. Bars with a different letter within individual clusters are significantly different (one-way ANOVA followed by Tukey post hoc test, *p* < 0.05). Letters in the order of increasing hemostatic activity: (a), (b), (c), (d).

Methanol extracts of *C. megalocarpus* (H.), *L. camara* (L.), and *C. megalocarpus* (H.)-*L. camara* (L.) blend were found to have observable protein precipitation activity. This was indicated by the higher relative astringency (RA) compared with that of the negative control as illustrated in Figure 2. However, at 0.25 and 0.5 mg/ml concentrations, methanol extracts of *C. megalocarpus* (H.) and *L. camara* (L.) were found to have significantly lower protein precipitation abilities compared with the reference drug (IvyZinc™) (*p* < 0.05; Figure 2). At 1 mg/ml concentration, the activity of methanol extract of *C. megalocarpus* (H.) contrasted with findings seen at lower concentrations where the effects were significantly higher than that of the reference drug (*p* < 0.05; Figure 2). The protein precipitation abilities of the three plant extracts were found to be dose-dependent, where the largest dose had a significantly higher relative astringency compared with lower doses (*p* < 0.05; Figure 2).

**Figure 3:**
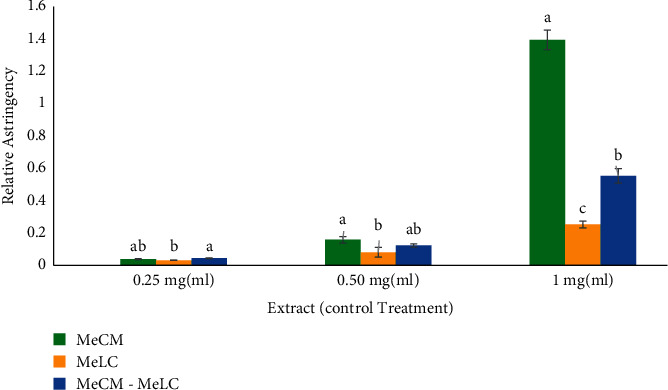
Comparison of relative astringency effects of MeCM, MeLC, and MeCM-MeLC extracts. MeCM (methanol leaf extract of *Croton megalocarpus*), MELC (methanol leaf extract of *Lantana camara*), and MeCM-MeLC (methanol *Croton megalocarpus* and *Lantana camara* blended leaf extract). Each bar graph in the respective group represents 5 separate sample runs (*n* = 55) and is expressed as the mean ± SEM of the dataset. Error bars represent the standard error of mean measurement. Bars with a different letter within individual clusters are significantly different (one-way ANOVA followed by Tukey's post hoc test *p* < 0.05). Letters in order of increasing hemostatic activity: (a), (b), and (c).

Compared to each other, the relative astringency of the methanol extract of *C. megalocarpus* (H.) and that of the *C. megalocarpus* (H.)-*L. camara* (L.) blend was not significantly different at a concentration of 0.25 mg/ml (*p* > 0.05) as shown in Figure 3. However, the activity of *L. camara* (L.) extract, at a concentration of 0.25 mg/ml, was significantly lower (*p* < 0.05) than the effect of the *C. megalocarpus* (H.)-*L. camara* (L.) blend (Figure 3). *C. megalocarpus* (H.) extract at a concentration of 0.5 mg/ml was found to have a significantly higher protein precipitation activity compared with the effect of *L. camara* (L.) (*p* < 0.05) at a similar concentration. However, the effect of the *C. megalocarpus* (H.)-*L. camara* (L.) blend extract was not found to be significantly different from the effects of individual plant extracts at concentrations of 0.25 and 0.5 mg/ml (*p* > 0.05; Figure 3). The relative astringency of the methanol extract of *C. megalocarpus* (H.), at a concentration of 1 mg/ml, was found to be significantly higher compared with that of *L. camara* (L.) and that of *C. megalocarpus* (H.)-*L. camara* (L.) blend at a similar concentration (*p* < 0.05) as illustrated in Figure 3. The methanol extract of *C. megalocarpus* (H.)-*L. camara* (L.) blend at a concentration of 1 mg/ml caused a significantly higher protein precipitation activity compared with that of the *L. camara* (L.) at the same concentration (*p* < 0.05; Figure 3).

Phytochemical analysis: methanol extract of *Croton megalocarpus* Hutch contained reducing sugars, cardiac glycosides, flavonoids, tannins, steroids, terpenoids, phenols, and carbohydrates. During preliminary phytochemical evaluation, *Lantana camara* Linn leaf extract contained cardiac glycosides, phenols, carbohydrates, saponins, reducing sugars, terpenoids, and tannins.

## 4. Discussion

The maintenance of blood in a fluid state depends on a preserved vascular endothelium [2]. When this is breached, primary hemostasis ticks in with Von Willebrand factor (vWF) binding to collagen on the exposed endothelium to release thromboxane A2 and triggers the release of adenosine diphosphate (ADP). Thromboxane A2 causes immediate vasoconstriction, thereby controlling blood loss mechanically [3]. Together with ADP, thromboxane A2 attracts and activates ambient platelets which bind to vWF, forming a plug on the bleeding site [2].

Secondary hemostasis follows, where a series of sequential activation steps of enzyme precursors (zymogens) to active proteases in a cascade fashion result in the generation of thrombin [3]. The cascade takes place on three pathways: intrinsic, extrinsic, and common. Thrombin converts soluble fibrinogen into fibrin which forms over the platelet plug, providing reinforcement [2]. Precipitation of proteins *in vitro* leads to thromboxane A2 synthesis, which promotes platelet aggregation and acceleration of the formation of the temporary platelet plug over the injury site [17]. Materials from the leaves of *C. megalocarpus* (H.) and *L. camara* (L.) are applied topically to control peripheral bleeding [10, 11]. In the current study, the activities of extracts from both plants were evaluated for their ability to precipitate hemoglobin, the red blood cell protein. The hemoglobin molecule has a quaternary structure, with four polypeptide chains held together by covalent bonds, a heme group each, bearing a single ferrous ion [18]. When compared to the negative control group, the methanol extracts of *C. megalocarpus* (H.), *L. camara* (L.), and the *C. megalocarpus* (H.)-*L. camara* (L.) blend demonstrated higher protein precipitation activities. This was indicated by the significantly higher relative astringency recorded at all extract concentrations tested. Interestingly, the methanol extract of *C. megalocarpus* (H.) showed higher protein precipitation ability compared with that of*L. camara* (L.) across all the three tested extract concentrations evaluated.

In a similar study involving *Tridax procumbens* leaf extract, it was found to have hemoglobin precipitation abilities [19]. Extracts of *Pimpinella battandieri* had the ability to precipitate hemolyzed blood from bovine samples [20]. An aqueous extract of *Pituranthos scoparis* bark precipitated hemoglobin from blood sample hemolysates [21]. In separate studies, extracts of *Phlomis bovei* De, *Hertia cleirifolia*, *Cucumis melo*, and *Myrtus communis* precipitated proteins in bovine blood [22–25]. Previous studies have linked plant and animal-derived enzymes (proteases) and phytochemicals of plant origin, especially phenolics, terpenoids, and organonitrades, to protein precipitating ability. Falcipain, a proteolytic enzyme produced by the *Plasmodium falciparum* parasite, is associated with the hydrolysis of hemoglobin into amino acids for reuse in protein synthesis [26]. Proteolytic enzymes isolated from the *Vallesia glabra* plant hydrolyze peptide bonds of hemoglobin to release polypeptides and amino acids [27].

However, it should be noted that extracts from both *C. megalocarpus* (H.) and *L. camara* (L.) were not found to contain proteins during the preliminary phytochemical screening. The observed protein precipitation effects of the studied extracts in this study could be attributed to the phytochemical constituents. Phytochemicals containing phenolic rings, namely, lignans, tannins, flavonoids, and phenols, have been shown to be associated with protein precipitation properties [19, 23]. Tannins and phenols present in aqueous extracts of *Hertia cleirifolia* are associated with its ability to precipitate bovine blood proteins [22]. A study involving the *Piper betle* attributed the presence of tannins found in the extract to its protein precipitation activity [28]. Tannins lyse cells in soft tissues to expose proteins which are later precipitated [17].

However, it was pointed out that the role of tannins in bleeding control is partial; while working with *Tridax procumbens*, tannins were associated with protein precipitation activity that requires complementation by other mechanisms [18]. Tannins like ()-epicatechin-3-O-gallate or ()-epigallocatechin-3-O-gallate cause precipitation of gelatin, a protein, *in vitro*. Tannin's molecules were also demonstrated to interact and precipitate proteins, as well as polysaccharides and fellow polyphenols like flavonoids [29]. In a study on the effects of saponins on human erythrocytes, it was found that the phytochemicals hemolyzed cells in blood samples [17]. Flavan-3-ol, a phenolic flavonoid, has been linked to the precipitation of salivary proline-rich protein [30]. Lignans (polyphenols) purified from Opuntia seeds were found to precipitate proteins due to their structural flexibility and polarity [31].

In a study based on plant-derived Agrimooni, it was noted that tannins cause precipitation by a mechanism that leads to the formation of cross-links with the protein molecule [32]. Phenolics (including tannins and phenols) establish cross-links with proteins involving hydrogen bonds between the hydroxyl groups of the phenolic group and the carbonyl groups on the amino ends of the proteins [33]. The interaction between some flavonoids and lactoferrin (milk proteins) results in binding via van der Waals forces and hydrogen bonds [34].

Saponins form complexes with proteins, preserving their structure from denaturation [35]. Saponin's aglycone moiety has an affinity for red blood cell wall membrane sterols [35]. Their binding with fragments of red cells and eventual deposition upon centrifugation is postulated to cause an increased mass within the complexes, thereby resulting in deposition during centrifugation. At concentrations of 0.25 and 0.5 mg/ml, the methanol extract of *C. megalocarpus* (H.)-*L. camara* (L.) blend had comparable activity to separate extract of *C. megalocarpus*. However, the activity of *C. megalocarpus* (H.)-*L. camara* (L.) blend methanol extract was lower than that of *C. megalocarpus* (H.) at 1 mg/ml concentration. When tannins are mixed with proteins, the precipitates formed disperse in solution to saturation and may be reversed by the addition of more tannins [36]. This explains why the relative astringency of the methanol extract of *C. megalocarpus* (H.)-*L. camara* (L.) blend extract is lower than that of the separate *C. megalocarpus* (H.) extract at 1 mg/ml concentration.

## 5. Conclusion

This study revealed that methanolic extracts of *C. megalocarpus* (H.) and *L. camara* (*L*) have red blood cell protein precipitation qualities. Protein precipitation qualities are postulated to support the physiology of primary hemostasis through mediating vasoconstriction following vascular injury. This finding validates the continued use of materials from both plants in peripheral bleeding control. The study recommends further research on fractions of *C. megalocarpus* (H.) and *L. camara* (*L*) on wound healing qualities and scar tissue histology. Extracts from both plants are potential candidates for drug development.

## Data Availability

The quantitative and qualitative data used to support the findings of this study are included within the article. Additional information on raw can be obtained from the corresponding author upon request.
